# The Effects of Exercise Intervention in Older Adults With and Without Sarcopenia: A Systematic Review

**DOI:** 10.3390/sports13050152

**Published:** 2025-05-19

**Authors:** Jeremy Cabrolier-Molina, Alexandra Martín-Rodríguez, Vicente Javier Clemente-Suárez

**Affiliations:** 1 Faculty of Medicine, Health and Sports, Universidad Europea de Madrid, Villaviciosa de Odón, 28670 Madrid, Spain; jeremoc@gmail.com (J.C.-M.); vctxente@yahoo.es (V.J.C.-S.); 2 Faculty of Education Sciences, International University of Business (UNIE), 28015 Madrid, Spain; 3 Grupo de Investigación en Cultura, Educación y Sociedad, Universidad de la Costa, Barranquilla 080002, Colombia

**Keywords:** aging, geriatric exercise, muscle function, fall prevention, functional capacity, balance, risk

## Abstract

This systematic review, conducted in accordance with PRISMA guidelines and registered in PROSPERO (CRD42024619693), aimed to evaluate the effects of physical exercise interventions on muscle function and fall risk in older adults with and without sarcopenia. **Methods**: A comprehensive search of PubMed and Web of Science databases identified 11 randomized controlled trials (RCTs) published between 2015 and 2025. A total of 792 participants (mean age 75.13 ± 4.71 years; 65.53% women, 34.47% men) were included. Interventions varied in type—strength, balance, aerobic, and multi-component programs—with a minimum duration of 8 weeks. **Results**: The reviewed studies showed that physical exercise interventions significantly improved neuromuscular function, physical performance, and postural control in older adults. Positive effects were observed in gait speed, stair-climbing ability, grip strength, muscle mass, and bone density. Specific modalities such as Tai Chi improved postural control and neuromuscular response; dynamic resistance and functional training increased muscle strength and improved posture; Nordic walking reduced postural sway; and multi-component and combined walking-resistance training enhanced mobility and force efficiency. Programs integrating strength and balance components yielded the most consistent benefits. However, reporting on FITT (Frequency, Intensity, Time, Type) principles was limited across studies. **Conclusions**: Exercise interventions are effective in improving neuromuscular outcomes and reducing fall risk in older adults, both with and without sarcopenia. The findings support the need for tailored, well-structured programs and greater methodological standardization in future research to facilitate broader clinical application and maximize health outcomes.

## 1. Introduction

With increased life expectancy due to advances in medicine, access to essential services, and better nutrition, the elderly population has grown significantly. This rise presents a challenge for global healthcare systems, as aging is linked to a higher risk of falls. The WHO predicts that between 2015 and 2050, the global population over 60 will double from 12% to 22%, with 2020 marking a higher percentage of people in this age group than children under five [1]. In Europe, indicators recorded over the past decades show that out of the 11.7 million medical care requests made by people over the age of 65, 8.4 million are related to injuries caused by falls, with 54,504 deaths documented as a result. Additionally, the incidence rate of fall-related injuries requiring medical attention is higher in women than in men [2].

Aging is commonly associated with a progressive decline in physical function, particularly due to sarcopenia which contributes significantly to reduced mobility and increased fall risk. Sarcopenia is the term used to describe significant changes in body composition and function as a consequence of muscle mass loss related to disease or the normative aging process [3]. It may also be accelerated by various factors, including physical inactivity, poor nutrition, and inflammation due to chronic diseases [4].The European Working Group on Sarcopenia in Older People (EWGSOP) defines sarcopenia as a muscular disease or syndrome characterized by the progressive and generalized loss of muscle mass and strength, with a risk of adverse outcomes such as physical disability, poor quality of life, and death [5]. The condition is further complicated by variations in its classification (e.g., acute vs. chronic, or sarcopenic obesity) and the absence of universally accepted diagnostic criteria or standardized tools to assess muscle mass and function. This lack of consensus presents a significant barrier to clinical translation and the design of effective interventions.

In older adults diagnosed with sarcopenia, the loss of muscle mass and strength does not occur uniformly. Instead, it affects specific muscle groups to varying degrees, with some regions demonstrating significantly greater vulnerability to age-related atrophy. Most research to date has focused primarily on large muscle groups, particularly in the lower limbs, such as the quadriceps femoris, which have consistently been shown to undergo marked degeneration with age. Studies have reported reductions in quadriceps muscle size ranging from approximately 0.53% to as much as 27% per year, depending on the individual’s activity level, nutritional status, and presence of comorbid conditions. This deterioration significantly impairs mobility, impedes the ability to perform everyday tasks, and increases the risk of falls [3].

However, focusing solely on the major muscle groups of the hips and thighs in training interventions is insufficient when the goal is to prevent falls and improve postural control in the elderly. Increasing attention must be given to smaller but functionally critical muscles, particularly those of the feet and lower legs [3,4]. The intrinsic and extrinsic muscles of the foot, such as the flexor digitorum longus, flexor hallucis longus, and tibialis posterior, are essential for maintaining balance and supporting dynamic stability during gait. These muscles contribute to the fine adjustments necessary for postural reactions and adaptive movements on uneven terrain. Additionally, the peroneal (fibular) muscles—peroneus longus and brevis—play a pivotal role in lateral stabilization of the ankle, preventing inversion sprains and contributing to overall gait efficiency [4,5].

Neglecting these muscle groups in both assessment and intervention strategies may result in an incomplete approach to fall prevention and mobility enhancement. Therefore, comprehensive exercise programs for older adults should not only address large muscle groups but also incorporate exercises that specifically target these smaller stabilizing muscles to maximize functional outcomes and reduce the incidence of falls. Future research should further explore the role of these under-studied muscles in age-related decline and their potential for functional rehabilitation through targeted training [6].

However, there is still a lack of consensus on operational and diagnostic definitions, possibly due to the absence of tools that accurately measure elements of interest, such as skeletal muscle rather than lean mass, many research findings have yet to be translated into clinical practice [5]. Given the diversity of causes associated with this condition, several types of sarcopenia have been distinguished. This classification differentiates between primary sarcopenia, related to aging, and secondary sarcopenia, related to diseases. It also categorizes sarcopenia into acute and chronic forms, with the former lasting six months or less and the latter lasting more than six months. Both forms share the characteristic of originating from diseases or injuries; however, chronic sarcopenia is associated with chronic and progressive medical conditions that increase the risk of mortality. Lastly, sarcopenic obesity is the reduction of muscle mass in the context of increased adiposity, prevalent in older adults, and the risk of developing it increases with age [7].

Despite the lack of consensus, the existing evidence is clear about the benefits of physical activity, and even more so of exercise, as a treatment for at least 26 diseases and 65 other conditions [8]. It also serves as a form of prevention, improving health and physical condition, which, in this group of people, additionally reduces the risk of falls that can result in fractures and other physical injuries. These incidents increase fragility, reduce mobility, and can lead to greater loss of functionality and cognitive decline. In turn, this can cause dependency and significantly decrease the quality of life of those affected, as well as increase their morbidity.

This absence of standardized guidelines can be attributed to the heterogeneity of exercise protocols, participant characteristics, and outcomes across studies [5]. There is no universally agreed-upon approach regarding the optimal type, intensity, frequency, and duration of exercise needed to produce consistent and clinically meaningful improvements in older populations. This is largely due to the wide variability in study designs, populations, and outcome measures reported in the literature. In fact, Mikel Izquierdo et al. reported recently that the lack of standardized guidelines for physical exercise interventions stems from the complexity of tailoring exercise to the diverse needs and medical conditions of older adults. Specifically, the need for precise dose-response adaptations, and the integration of exercise as a medical treatment complicate the development of universal standards [8]. Concretely, a key barrier to the development of standardization lies in the complexity of adapting physical activity to the diverse health profiles and functional capacities of older adults. As highlighted in recent consensus publications, tailoring exercise prescriptions requires consideration of factors such as individual variability in response to training, comorbid conditions, and the need to integrate exercise into medical care. Moreover, many training programs fail to account for the specific neuromuscular demands of aging, such as the decline of fast-twitch muscle fibers (type II), particularly in lower limbs, which play a crucial role in preventing lateral falls and maintaining postural balance [8].

Due to this, the present systematic research addresses a clear research gap: the lack of consensus and standardized evidence regarding which types of exercise interventions—specifically in terms of strength, balance, aerobic, and multi-component training—are most effective in improving muscle function and reducing fall risk in older adults, particularly those with or without sarcopenia. While the benefits of physical activity are well established, the existing literature shows considerable heterogeneity in intervention types, durations, and participant characteristics, making it difficult to determine optimal training prescriptions [8,9,10,11,12]. The rationale of the review is to synthesize findings from randomized controlled trials (RCTs) to determine which of these approaches—or combinations thereof—yield the most significant improvements in strength, functional capacity, postural control, and fall prevention in the aging population. By clarifying these aspects, the review aims to support more precise and effective exercise prescriptions for clinical and community settings [13].

Training programs with exercises that focus on movement patterns are likely to be the most effective, as many of these movements, which are normal in people’s daily lives, tend to be abandoned as they age. This influences the specific muscle atrophy related to aging, and this disuse atrophy can also be observed in younger individuals who exhibit higher levels of sedentary behavior [9]. In the long term, this will be crucial in the likelihood of experiencing falls, especially lateral ones. This is primarily because sarcopenia is associated with the atrophy of type 2 muscle fibers, also known as fast-twitch fibers [10].

This systematic review synthesizes knowledge derived from several training programs evaluated [6]. The primary objective of this review is to evaluate the effects of physical exercise interventions on muscle function and fall risk in older adults with and without sarcopenia. Secondary objectives include examining improvements in functional capacity, physical condition, and overall mobility. Given this context, we hypothesize that exercise interventions incorporating functional, multi-directional movement patterns are more effective in improving muscle function and reducing fall risk than isolated or single-plane exercises, especially in populations with sarcopenia or those exhibiting sedentary behaviors.

By compiling these findings, this review facilitate access to effective training methods by concentrating and organizing relevant approaches for the design of training programs aimed at older adults. These programs should include a wide range of stimuli within their programming, offering more personalized and effective alternatives, thereby improving the health and quality of life of this population.

## 2. Materials and Methods

### 2.1. Search Strategy

This systematic review was conducted in accordance with the Preferred Reporting Items for Systematic Reviews and Meta-Analyses (PRISMA) guidelines and registered in the PROSPERO International Prospective Register of Systematic Reviews (ID: CRD42024619693). The aim was to evaluate the effectiveness of various exercise interventions on muscle function and fall risk in older adults with or without sarcopenia [14].

### 2.2. Literature Search Strategy

A systematic literature search was carried out in PubMed and Web of Science, selected for their comprehensive coverage of exercise science and aging-related research. The search strategy used Boolean combinations adapted for each database. The final base search expression was:

((“sarcopenia” AND “training”) AND (“elderly” OR “older adults”) AND “fall”)

The search included English-language articles published between 2015 and 2025. Non-English studies and grey literature were excluded.

### 2.3. Eligibility Criteria and Study Selection

Eligibility criteria were defined according to the PICO framework [15]:

**Population (P):** Adults aged ≥65, with or without sarcopenia.
**Intervention (I):** Physical exercise programs of ≥8 weeks duration.
**Comparator (C):** Alternative interventions or control groups.
**Outcome (O):** Muscle strength/function, fall risk reduction, and functional capacity.

### 2.4. Software

The filtering of articles for this systematic review was performed using the software RAYYAN (Rayyan Systems, Inc., Cambridge, MA, USA), a web-based tool designed to facilitate the selection and screening of references in systematic reviews. RAYYAN allows for greater efficiency and speed in the review of articles thanks to features such as color coding, smart filters, and collaboration options for teamwork. Concretely, two authors (J.C.M. and A.M.R.) screened titles and abstracts using this software, which facilitated blind screening and conflict resolution. In case of disagreement, a third author (V.J.C.S.) was consulted. Full texts of eligible studies were then reviewed.

### 2.5. Quality Assessment

The methodological quality of included studies was independently assessed by two authors using the Downs and Black checklist, a validated tool suitable for both randomized and non-randomized studies. The checklist evaluates 27 items across five domains: reporting, external validity, bias, confounding, and power. Scores were categorized as follows:

- Excellent (26–28 points)
- Good (20–25 points)
- Fair (15–19 points)
- Poor (<14 points)

Any discrepancies in scoring were resolved through discussion or consultation with the third author.

### 2.6. Inclusion/Exclusion Criteria Selection of Studies

**Figure 1:**
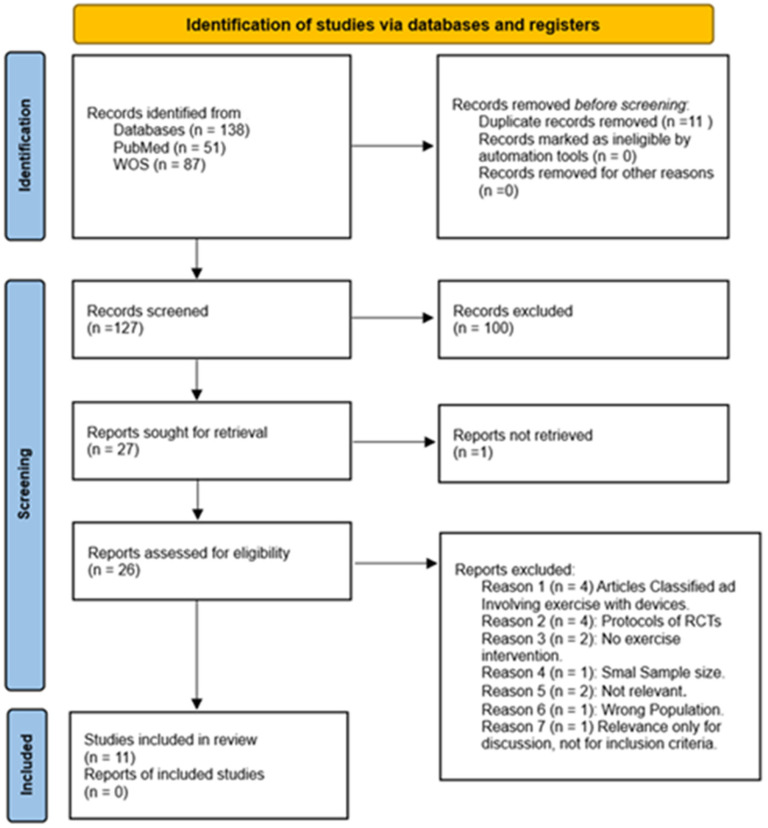
Flow diagram of study selection process.

To provide a clear overview of the selection process and enhance transparency, a PRISMA flow diagram is included (see Figure 1). This diagram represents the different stages of the review process, from the initial identification of articles to the final selection of studies. The diagram details the flow of information, describing the number of records identified, screened, and excluded, as well as the final studies included in the review. This figure helps to visualize the systematic approach used to filter the literature in this systematic review.

The screening process was conducted independently by two authors. In cases of disagreement regarding article inclusion, a third author was consulted to resolve the issue and reach a consensus.

Regarding the design of this systematic review, the reviewed literature was required to be no more than 10 years old, with search filters set to studies published from 2015 to 2025. The studies included in this systematic review involved adult participants within the age range categorized as “Older Adults,” with varying degrees of musculoskeletal functionality and health conditions, whether under medication or not at the time of participating in the intervention (Table 1). All selected articles were randomized controlled trials, due to their qualitative characteristics of rigor and reliable methodological quality [13] (Table 2).

These studies were conducted across various countries, including Germany, Hungary, Portugal, Finland, Indonesia, China, and the United States. Participants were recruited from a range of institutions and communities using diverse recruitment methods, such as posters, online advertisements, postal mail, and health campaigns. Specific recruitment sites included nursing homes, social care centers, and pensioner associations. In China, outpatients from the Zhe Jiang Hospital Department and surrounding communities were involved. German research focused on nursing home residents, and in the United States, older adults with a history of falls in the past year were studied. In Hungary, the sample consisted of older women with mild to moderate sarcopenia, and in Portugal, institutionalized octogenarians with low postural stability and high fall risk were examined. Participants in Indonesia were elderly with locomotive syndrome stage 1. Lastly, in Finland, the participants were community-dwelling older adults who did not meet physical activity guidelines.

The search process yielded 138 results. After duplicate identification, 11 were removed, leaving 127 articles for the selection process. During the initial screening phase, titles and abstracts were reviewed, leading to the exclusion of 100 articles that were not randomized controlled trials, leaving 26 articles for the second phase of screening review (see Figure 1).

The excluded articles are presented in detail below:

The 100 articles excluded were categorized as follows: Reviews and meta-analyses included systematic reviews or meta-analyses (11), reviews (23), narrative reviews (8), literature reviews (1), case reviews (1), and overviews (1). Protocols were categorized separately (3). Observational studies included observational studies (13), cohort studies (4), and observational cohort studies (3). Cross-sectional studies comprised both cross-sectional studies (8) and transversal studies (2). Experimental studies included quasi-experimental intervention studies (1), uncontrolled intervention studies (1), case studies (1), other experimental studies (2), prospective studies (1), and prospective longitudinal studies (1). Additional categories included animal research (1), genetic/genomic association studies (1), opinion statements (1), consensus statements (2), books (1), retracted publications (1), clinical commentaries (1), umbrella reviews (1), and scooping reviews (1). Articles classified as not related totaled (5).

In the second phase of screening, from the 26 studies that remained, 1 study was excluded due to paid access, and 15 articles were excluded after the initial review. The breakdown is as follows: Articles classified as involving exercise with devices (4), protocols of RCTs (4), no exercise intervention (2), not relevant (2), wrong population (1), and small sample size (1), Relevance only for discussion, not for inclusion criteria (1). This left 11 studies that met the necessary criteria for inclusion in this systematic review.

## 3. Results

The 11 studies selected for this systematic review evaluated the effects of physical exercise interventions on functionality and fall risk in older adults. In total, 792 participants (75.13 ± 4.71 years) were included across the studies, comprising 519 women (65.53%) and 273 men (34.47%). In most studies, the proportion of female participants was higher or exclusively female. The interventions encompassed a wide range of exercise modalities, intervention durations, and sample characteristics, reflecting the heterogeneity commonly observed in this population. To provide a clearer understanding of the characteristics and dose-response relationships of the interventions, we have systematically incorporated the FITT principles (Frequency, Intensity, Time, and Type) into our data extraction and summary. The results consistently indicated significant improvements in muscle strength, functional capacity, and reductions in fall risk. A detailed summary of the interventions and their characteristics is presented in Table 2, Table 3 and Table 4.

In this study, the positive effect of physical exercise on the body was observed through various interventions. In these, the baseline parameters showed significant improvements in the functionality of older adults. A detailed description of the 11 studies is provided detailing the methods and findings of each intervention, the aim is to offer a synthesized view of the recorded data that allows for the identification of variables and changes in their parameters, providing a deeper understanding of the effectiveness of different exercise modalities in muscle strengthening and fall risk reduction.

Various measurement instruments were used to evaluate different aspects of physical performance and health across these studies. Walking speed and time on the TUG test were recorded, alongside strength evaluations using the chair rise test, maximum strength tests with a leg press machine, and dynamometers., along with muscle mass and its. The body composition was analyzed via bioimpedance and bone densitometry (DXA), Handgrip strength was measured with dynamometer [13]. Muscular strength was further assessed using electromyography (EMG) in key muscles such as the rectus femoris, semitendinosus, tibialis anterior, and gastrocnemius electromyography [14,15].

Balance was assessed through specific postural control tests that evaluated mobility, spinal deformities, and overall stability under various conditions (e.g., eyes open and closed, wide and narrow stances) using force platforms and validated questionnaires. To assess postural control [23], the Dynamic Stability Test (ProKin 254) was used, and the Overall Stability Index (OSI) [15] was measured. The incidence of falls and fear of falling were monitored through questionnaires and follow-up calendars [18]. Adherence and participant viability were also measured, and cognitive tasks were included to assess mental function. The level of sarcopenia was measured using the Sarcopenia Mass Index (SMI) [14].

The studies focused on evaluating the effect of a physical exercise program on the elderly population. The most common results from the reviewed studies included improvements in muscular strength, specifically in the lower extremities, and a reduction in the incidence of falls. Significant improvements were reported as a result of the interventions, with increases observed in the experimental groups and decreases in the control groups. Assessments such as the Timed Up and Go (TUG) test and strength tests indicate improvements in physical condition. Another observed improvement was a small but significant change in lean mass and a reduction in fat mass, particularly in the legs. These changes in body composition have been associated with improvements in mobility and functionality, reflecting a positive impact on overall health [16,20]. Regarding postural control and the incidence of falls, improvements were observed that reduced the occurrence of falls, with some studies reporting zero falls in the intervention groups. Furthermore, there was a reduction in the fear of falling, which increased confidence among participants in the intervention group.

## 4. Discussion

Throughout this systematic review, quantitative data was analyzed from interventions that evaluated the effect of physical exercise from various perspectives and types of physical exercise in older adults. The main objective of this review was to evaluate the effects of physical exercise interventions on muscle function and fall risk in older adults with and without sarcopenia. The secondary objectives focused on examining improvements in functional capacity, physical condition, and overall mobility.

To address the variability between interventions, we have systematically analyzed and presented the key characteristics of each training program following the FITT criteria (Frequency, Intensity, Time, and Type). The interventions reviewed showed notable differences in frequency, ranging from two to seven sessions per week, and in the duration of each session, generally between 30 and 60 min. Training intensities were adjusted depending on the objectives of each program, using methods such as percentages of maximal strength (e.g., 1RM or 8RM), perceived exertion scales, or heart rate monitoring, adapting the load according to the participants’ capacities. The types of exercise applied were diverse, including strength training with machines or elastic bands, Nordic Walking at different intensities [8,10], multicomponent programs that combined strength, balance, and aerobic work, and mind-body approaches like Tai Chi [15]. This structured extraction allows us to better interpret the dose-response relationship of each intervention, considering that higher intensities and frequencies tend to generate greater improvements in muscle mass, functional capacity, and reduction of fall risk, although moderate and tailored interventions may be more suitable for older adults with frailty or comorbidities. Including this detailed analysis based on FITT variables strengthens the conclusions drawn and highlights the importance of individualized, well-planned exercise interventions in older populations.

Given the diversity of approaches identified, we now proceed to describe in greater detail the most relevant aspects related to the specific variables analyzed across the different studies.The reviewed interventions were based on different methods, utilizing various types of stimuli, such as those focusing on breathing and body alignment to improve balance and health based on gentle and fluid movements that combine principles of meditation and martial arts, as is the case with Tai Chi [15], the use of hypoxia [18] or training programs based on different intensities [23]. The intensity of the exercise plays a crucial role: high-intensity programs, such as strength training, tend to induce more pronounced physiological adaptations, improving muscle mass and strength compared to lower-intensity interventions. [27]. Nevertheless, these high-intensity interventions may not be suitable for all older adults, particularly those with comorbidities or pre-existing conditions.

The duration of the intervention is also a determining factor in effectiveness; longer interventions tend to show greater improvements in functionality and reduced fall risk. However, this can also affect adherence, especially if participants experience fatigue or pain. Furthermore, the target population is a relevant factor: certain programs may be more effective for specific groups, as physical stimuli have a different impact depending on health status, age, and previous activity level.

Overall, the improvements in the parameters studied in different groups of older adults aged 65 and above, with diverse physical and health conditions, such as men with osteosarcopenia [16], women with sarcopenia [20], or with locomotor syndrome [13], indicate that these interventions have had positive effects.

These interventions resulted significant improvements in the functional capacity of older adults, including an increase in muscular strength, cardiorespiratory capacity, coordination, and balance, as well as a decrease in the risk of falls [24]. Significant improvements were observed in the Sarcopenia Mass Index (SMI), compared to baseline measurements [14], this suggests that exercise performed with a well-designed program can serve as a tool to address sarcopenia with the goal of maintaining mobility and reducing the risk or impact of chronic diseases associated with aging. This process brings about a morphological change in older adults, decreasing functionality due to sarcopenia, which involves not only the loss of muscle mass, primarily of fast-twitch fibers, but also the impairment of their nerve connections, resulting in a loss of motor control [28], thereby increasing the risk of falls. Among the most common mechanisms of falls in the elderly are lateral and forward falls. Hip fractures are the most frequent in this population, followed by fractures of the femur and humerus [26]. The positive results obtained from strength training programs indicate their usefulness in improving functionality [19] postural control, and the reduction of kyphosis and hyper kyphosis. This condition is characterized by excessive curvature of the thoracic spine, which causes a hunched posture forward. Improving strength reduces this condition, thereby decreasing the risk of falls in octogenarians within the first month of training [17]. Interventions with entirely different training programs, such as those based on Nordic Walking, have also shown significant benefits. This type of exercise involves coordinated movement of both body hemispheres and improves stability. In this study, two training programs were developed: one at high intensity and the other at moderate intensity, with a volume of 120 min per session and a frequency of three times a week, supplemented with vitamin D. In the comparative analysis of data at the end of the intervention, improvements were observed in postural control and muscle mass in the lower limbs, suggesting that both variables may contribute to the positive results observed [23]. These improvements in strength gained through training not only contribute to increased muscle mass but also enhance postural balance control. This, in turn, improves coordination and balance, increasing stability, which may reduce the risk of falling, one of the main concerns of the population as they age [29].

Globally, it is expected that the proportion of older adult women will increase even further in the coming decades, according to WHO estimates [30]. Given that women 908 (61.0%) constitute more than half of the studied population, those studies can be useful tools to develop and evaluate training programs that consider the specific needs and characteristics of older women [31]. The results of this review suggest that addressing sarcopenia through physical exercise interventions can lead improvements in body composition and muscle strength, but also contribute to better posture, which could eventually reduce the likelihood of falls in the female population.

The heterogeneity of the studies presents a significant challenge for the generalization of the results. indicating the wide range of variables that interact with human functionality. The differences in the duration of interventions, types of exercise (such as strength training, Tai Chi, Nordic Walking, etc.), intensity of the programs, and characteristics of the studied populations (including age, physical status, and presence of comorbidities) contribute to the variability in results [8,10]. This makes it difficult to extrapolate the observed effects to a broader population. For instance, some studies included healthy older adults, while others focused on individuals with sarcopenia or pre-existing conditions, which could explain the differences in the effects observed on muscle strength, mobility, and fall risk. Moreover, the lack of standardization in measurement methods (such as differences in how muscle strength or fall risk are assessed) further limits the ability to directly compare results across studies [8]. These limitations highlight the need for more standardized research that addresses these variations and their impact on the effectiveness of exercise programs.

Additionally, randomized controlled trials aimed at evaluating the effects of physical exercise in populations with sarcopenia is limited. The medical community faces challenges in establishing criteria for diagnosing the levels of sarcopenia in older adults [24,32]. The lack of detail on the severity level of sarcopenia in study participants at baseline makes it difficult to generalize the findings, as they do not indicate whether the effects are observed in healthy older adults or those with defined levels of sarcopenia [20]. However, the findings offer valuable insights for designing exercise programs targeting sarcopenia, focusing on reducing its negative impact on the quality of life through interventions such as resistance training, strength training, or multi-component exercises [33]. While the results are promising, it is important to note the variability of the studies regarding the duration of interventions, the intensity and type of exercise, as well as the characteristics of the studied population (age, prior physical activity level, and presence of comorbidities). This variability complicates the generalization of the results and emphasizes the need for more standardized exercise guidelines for older adults. The limitations underscore the importance of further research to address these variations and improve the effectiveness of exercise programs.

Considering the multifaceted nature of older adult health, for the optimization of an exercise program, a multidisciplinary approach is ideal, as understanding other variables that influence the functionality of older adults will enrich the program’s design. When designing exercise programs, the context of each person must be considered to tailor the program according to their specific needs. These programs must include a variety of components, which must also be individualized. Strength training should control the loads using methods such as recording the speed at which the movement is performed, known as Velocity Based Training (VBT), and Effort Character (EC), which determine the intensity of each exercise. This allows differentiation of the intensity that the same load represents for two different individuals [34], as everyone has a different level of strength, motor control, previous experiences, and attitude toward strength training [35]. Cardiorespiratory training should be monitored through heart rate, using the appropriate intensities for each zone, in accordance with the effects these have on the body. It is essential to adjust the intensity zones based on specific physiological responses, such as improvements in aerobic capacity, anaerobic threshold, or cardiovascular efficiency, thereby optimizing the benefits of training for each individual [36]. In addition to these variables, it is important to incorporate exercises that enhance motor control, agility, balance, and reaction time [37]. Aging not only limits the body’s ability to produce strength, cardiorespiratory efficiency, or proprioceptive capacities, but also sensory abilities, as is the case with hearing. Although a higher level of muscular fitness and performance is associated with a lower incidence of hearing loss [38], the older adult population presents high rates of sedentary behavior [39]. For this reason, if the goal of a training program is to reduce the risk of falls, it should consider sensory capacities and their functional state. Hearing loss may be one of the main factors affecting balance in older adults [40,41]. Therefore, incorporating strategies to assess and address sensory deficits can enhance the overall effectiveness of exercise interventions. These adaptations can help reduce the risk of falls; however, they do not completely prevent them [42]. Therefore, it is necessary for training programs to include techniques that teach older adults how to react in the event of a fall, as this could make a significant difference in reducing the impact and severity of injuries when such situations occur [43].

### Limitations an Future Perspectives

One important limitation of this review is the lack of detailed information regarding the socioeconomic status and cultural or racial background of participants in the included studies. These factors can significantly influence older adults’ access to exercise programs, their adherence to interventions, and their perceptions of physical activity. The absence of this data limits our ability to analyze potential disparities in outcomes related to social or cultural determinants of health. Future research should aim to include and report these variables systematically to better understand their role in the effectiveness and accessibility of exercise interventions in diverse older populations.

Future research should prioritize the design and implementation of multicentre studies and those with greater methodological standardization. Multicentre studies can help address the variability in participant characteristics, environmental factors, and healthcare practices, thus improving the generalizability of findings across diverse populations [44,45,46,47,48].

**Figure 2:**
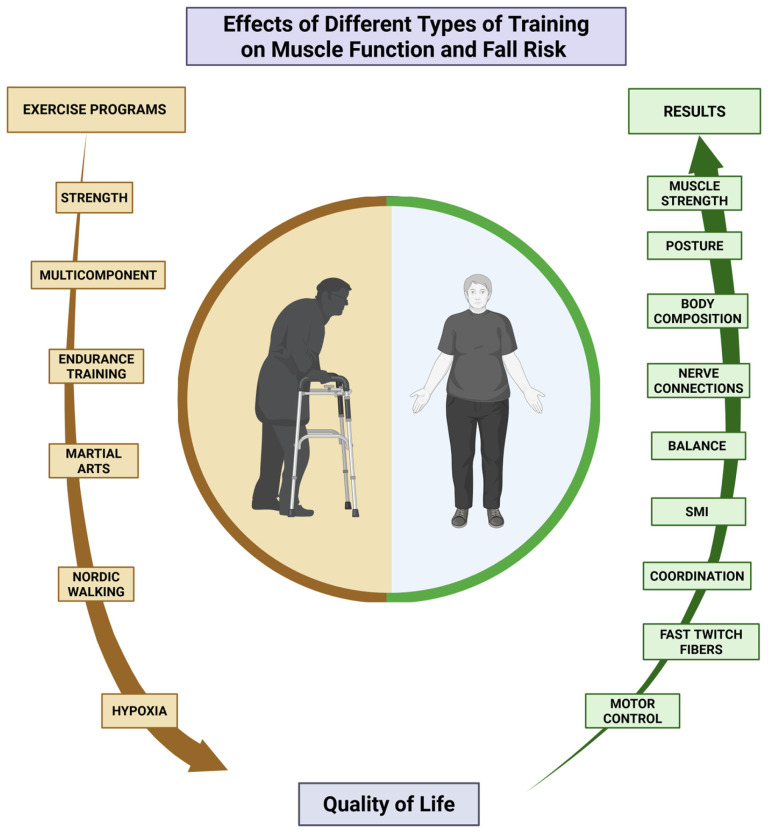
Summary of the results of the studies in terms of improvements in quality of life.

In summary, the interventions included in this study suggest that physical exercise is effective in improving functionality, increasing strength and muscle mass, and reducing the risk of falls in older adults (Figure 2) [44,45,46]. The results confirm that any form of physical activity can significantly improve the quality of life for this population. In addition to exercise programs having a multicomponent approach [49,50], it is crucial that they are led by qualified professionals, particularly sports science experts, to maximize long-term effects and adherence. This is especially important for promoting healthy aging [45], it is essential that they are guided by an expert in physical exercise, specifically a qualified sports science professional, to maximize their long-term effects and adherence. This is especially important for promoting healthy aging [44,45,46] which allows for maintaining personal independence, particularly in older adults between 65 and 75 years of age. For individuals beyond this age range, the focus should shift to maintaining mobility and preventing frailty, as they are at a higher risk of functional decline and dependence. This shift is crucial for preserving autonomy and minimizing fall risks among older adults.

## 5. Main Findings

The reviewed studies highlight the positive effects of various exercise interventions on neuromuscular function, physical performance, and postural control in older adults. For instance, Tai Chi training, as evaluated by Huang el at. [16] significantly improved postural control, measured through the Overall Stability Index (OSI), and enhanced neuromuscular response times. Similarly, machine-based and free-weight strength training, investigated by Johnen et al. [17], led to increases in gait speed, stair-climbing ability, and grip strength. Dynamic resistance training, examined by Kemmler et al. [18], resulted in gains in muscle mass and maintenance of bone mineral density. Multi-component exercise programs, assessed by LaStayo et al. [19], improved participants’ balance confidence and mobility, although no significant differences were observed between the intervention groups. Nordic Walking combined with vitamin D supplementation, analyzed by Mieszkowski el at. [20], effectively reduced postural sway, particularly in high-intensity training groups. Functional training programs, as studied by Minnet et al. [21], significantly enhanced muscle strength, posture, and body composition. Finally, combined walking and resistance training interventions, evaluated by Minett et al. [22], improved muscle function and force efficiency, further supporting the crucial role of structured exercise programs in mitigating aging-related physical decline.

### 5.1. Practical Applications

These programs can be implemented and adapted in real-life settings such as health centers, nursing homes, and community-based initiatives. For them to be effective and sustainable, several key aspects should be considered:

- Training for non-specialist professionals: Healthcare workers who engage with older adults—such as nurses, general practitioners, and caregivers—should receive specific training in exercise prescription. This should include knowledge of exercise types, intensity management, progression, and adaptation for common conditions such as sarcopenia, frailty, or chronic diseases.
- Personalization of interventions: Programs must be tailored to the individual characteristics of each older adult, considering not only physical capacity but also cognitive function, comorbidities, and sensory deficits (e.g., vision or hearing impairments).
- Multidisciplinary approach: Collaboration between sports scientists, physiotherapists, physicians, and social workers is essential to ensure a comprehensive and integrative strategy that goes beyond physical function and addresses overall well-being.
- Monitoring and motivation: Regular assessment of adherence, progress, and satisfaction—combined with motivational strategies—will help maintain engagement and long-term benefits.
- Infrastructure and accessibility: Exercise programs should be designed considering the environmental, economic, and logistical realities of the target population to ensure accessibility, equity, and inclusion.

### 5.2. Future Research Approach

- Conducting multicenter trials with standardized protocols to improve the generalizability of results.
- Investigating long-term outcomes, such as reduced hospitalization rates or preserved autonomy.
- Exploring the cost-effectiveness of implementing these programs in primary care or community settings.
- Evaluating the role of digital tools (e.g., wearable sensors or mobile apps) to support supervision, feedback, and remote interventions for older adults with mobility or geographic limitations.

## 6. Conclusions

This systematic review aimed to evaluate the effects of physical exercise interventions on muscle function and fall risk in older adults with and without sarcopenia. The findings show that physical exercise interventions led to significant improvements in muscle strength, postural control, balance, and mobility, as well as increases in muscle mass and reductions in the incidence and risk of falls. Improvements were observed in postural stability indicators, gait speed, stair-climbing ability, grip strength, and body composition, along with enhancements in neuromuscular response times and force efficiency. Multi-component programs also demonstrated a positive impact on balance confidence and mobility, while interventions such as Nordic Walking combined with vitamin D supplementation effectively reduced postural sway. Functional training further enhanced posture and muscular strength, supporting the crucial role of structured exercise in mitigating the effects of aging.

To strengthen the interpretation of the results, we have systematically incorporated the analysis of FITT principles into the data extraction process. This addition highlights the diversity in exercise program designs, ranging from strength training and Nordic Walking to Tai Chi and multicomponent interventions, and facilitates a clearer connection between intervention characteristics and clinical outcomes.

Moreover, the integration of physical exercise into healthcare practices remains insufficient. Greater collaboration among healthcare professionals, exercise specialists, and policymakers is essential to ensure that evidence-based exercise programs become a core component of preventive and therapeutic strategies for aging populations.

In conclusion, well-designed exercise interventions grounded in structured principles such as FITT represent an effective and cost-efficient strategy to enhance health, maintain functional independence, and improve quality of life in older adults. Addressing current gaps through standardized, individualized, and multidisciplinary approaches will be critical to maximizing the benefits of exercise in aging societies.

## Figures and Tables

**Table 1:** Inclusion and Exclusion Criteria.

Inclusion Criteria	Exclusion Criteria
Randomized controlled trials	Interventions not related to physical exercise.
2. Exercise interventions.	2. Studies that are not randomized controlled trials (RCTs).
3. Elderly population	3. Studies with a sample size of fewer than 20 participants.
4. Interventions that include fall risk reduction training.	4. Study Protocols
5. Studies reporting data on fall records.	5. Population under 60 years of age.
6. Interventions with a minimum duration of 8 weeks.	6. Interventions using devices with physical exercise.
7. Studies that include or focus on sarcopenia, if applicable.	7. Systematic reviews and meta-analyses

**Table 2:** Main Characteristics studies.

Authors	Study Design (RCT) º	Sample Size	Age Range	*p*-Value	Sex	Population (*)
Huang, 2023 [16]	RCT	n = 56	Tai Chi: 69.70 ± 5.05 Control: 72.14 ± 4.79	*p* = 0.07	F = 25 M = 31	China
Johnen, 2018 [17]	Two-arm, single-blind, RCT	n = 29	MT 78.9 ± 9.11 FWT 89.0 ± 2.80	*p* = 0.001	F = 20 M = 9	Germany
Kemmler, 2020 [18]	RCT	n = 39	Exercise 77.8 ± 3.6 Control 79.2 ± 4.7	*p* = 0.262	M = 39	Germany
LaStayo, 2017 [19]	Four-arm RCT	n = 134	RENEW 76.59 ± 7.39 TRAD 75.59 ± 6.98	*p* = 0.42	F = 87 M = 47	United States
Mieszkowski, 2018 [20]	RCT	n = 42	69.02 ± 5.57	Not specified	F = 42	Germany
Mile, 2021 [21]	RCT	n = 35	ACEi: 66.17 ± 1.18 Control: 66.55 ± 1.29	Not specified	F = 35	Hungary
Minett, 2020 [22]	Pilot RCT	n = 85	WALK: 75.8 ± 3.15 W + Exercise: 77.1 ± 4.90	Not specified	F = 48 M = 37	Germany
Nayasista, 2022 [23]	RCT	n = 20	Treatment: 75.4 ± 4.88 Control: 72.3 ± 4.30	*p* = 0.149	_	Indonesia
Rodrigues, 2022 [24]	Prospective clinical RCT	n = 27	MSEG: 86.37 ± 3.59 Control: 87 ± 4	*p* = 0.589	F = 20 M = 7	Portugal
Timon, 2021 [25]	RCT	n = 54	**Control:** 70.55 ± 4.0 **NT:** 70.35 ± 3.3 **HT:** 68.46 ± 3.8	*p* = 0.508	F = 54	Spain
Turunen, 2022 [26]	RCT	n = 291	PTCT: 74.4 ± 3.9 PT: 74.5 ± 3.7	Not specified	F = 188 M = 103	Finland

º All included studies were randomized controlled trials (RCTs), with some variations in design (e.g., pilot RCT, single-blind RCT, multi-arm RCT). * China: outpatients from the Zhe Jiang Hospital Department; Germany: nursing home residents; United States: older adults with a history of falls in the past year; Hungary: older women with mild to moderate sarcopenia. Portugal: institutionalized octogenarians with low postural stability and high risk of falls; Indonesia were elderly with locomotive syndrome stage; Finland: community-dwelling older adults who did not meet physical activity guidelines Notes: n = Sample size; F = Female; M = Male.

**Table 3:** Summary of FIIT Variables in Included Studies.

Study	Frequency	Intensity	Type	Session Duration	Intervention Duration	Co-Intervention
Huang, 2023 [16]	3/week	Borg CR-10 ≤ 4 (low-moderate)	Tai Chi (multicomponent)	40 min	12 weeks	No
Johnen, 2018 [17]	2/week	MT: 50–80% 8RM/FWT: RPE 12	Strength (machines or dumbbells)	45–60 min	12 weeks	No
Kemmler, 2020 [18]	2/week	High: RM, nRM, drop sets, supersets	Strength (machines, periodized)	~45–60 min	≥24 weeks (estimated)	Supplementation
LaStayo, 2017 [19]	3/week	TRAD: 60–70% 1RM/RENEW: Borg 7–13	Multicomponent + strength (TRAD or RENEW)	60 min	12 weeks	No
Mieszkowski, 2018 [20]	3/week	MI-NW: 60–70% HR/HI-NW: intervals + 70% HR	Nordic Walking (continuous vs. HIIT)	60 min	12 weeks	No
Mile, 2021 [21]	2/week	TRX: moderate (individualized)/Cardio: 50–55% HRmax	Functional multicomponent (TRX, cardio, fitball, stretching)	55 min	24 weeks	No
Minett, 2020 [22]	WALK: 5/week, W + EX: 5/week	Moderate (walking)/progressive (strength and balance)	Aerobic/Functional multicomponent	30 min/day	12 weeks	Nutritional education
Nayasista, 2022 [23]	Aerobic: 7/week, Locomotor: 3/week	Aerobic: 40–70% HRmax/Locomotor: low-moderate	Multicomponent: aerobic + balance/functional strength	30–60 min approx.	8 weeks	No
Rodrigues, 2022 [24]	3/week	RPE 6–7 (moderate to moderately high)	Strength with bands + proprioceptive balance	~45 min	40 weeks	No
Timon, 2021 [25]	3/week	RPE 6–8, progressive with bands and kettlebells	Functional strength (bands + loads)	45 min	24 weeks	Hypoxia (HT group only)
Turunen, 2022 [26]	4–5/week (2 supervised + 2–3 home)	Moderate-high (6RM-based, progressive)	Multicomponent + cognitive training (PTCT)	~60 min	52 weeks (12 months)	Cognitive training (PTCT)

**Table 4:** Goals, measurements, and outcomes.

Authors	Goals	Measurement (Instruments)	Outcomes
Huang, 2023 [16]	To explore the effect of 12 weeks of Tai Chi on neuromuscular responses and postural control in elderly patients with sarcopenia.	EMG (Electromyography) Dynamic Stability Test (ProKin 254): Assessed postural control ability using a movable platform. Overall Stability Index (OSI): Measured overall stability during the dynamic stability test. (*)	Neuromuscular Response Times: In the Tai Chi group, significantly positive correlations were observed between changes in OSI (Postural Control Indicator) and changes in neuromuscular response times after 12 weeks (*p* < 0.05): Rectus femoris: r = 0.682 (*p* < 0.001), Semitendinosus: r = 0.488 (*p* = 0.007), Anterior tibialis: r = 0.757 (*p* < 0.001), Gastrocnemius: r = 0.767 (*p* < 0.001) OSI (Postural Control Indicator): decreased significantly in the Tai Chi group, indicating better postural control and lower fall risk (*p* < 0.05). Control Group: No significant correlations were observed between changes in OSI and neuromuscular response times in the control group (*p* > 0.05).
Johnen, 2018 [17]	To assess the feasibility of a machine-based versus free weight strength training program and its effects on physical performance in nursing home residents	Feasibility Eligibility rate, recruitment rate, measurement completion rate, loss to follow-up, adherence (attendance at sessions). Physical Performance TUG, 30-s CRT, 10-m Walk Test, 11-Step Stair-Climbing Test, grip strength. (*) Body Composition: Weight, height, BMI. Residents.	Physical Performance 10-m gait (m/s): Improved in both groups; Group 1 (39.2%), Group 2 (14.8%) (*p* = 0.050). Stair climbing (s): Improved in Group 1 (9.78%), decreased in Group 2 (−12.7%) (*p* = 0.168). Chair rise: Significant improvement; Group 1 (79.4%), Group 2 (122%) (*p* = 0.064). TUG (s): Improved in both groups; Group 1 (20.6%), Group 2 (16.4%) (*p* = 0.034). Grip strength right (kg): Slight increase; Group 1 (14.2%), Group 2 (45.2%) (*p* = 0.004). Grip strength left (kg): Improved; Group 1 (61.7%), Group 2 (52.1%). (*p* = 0.020). Feasibility Loss to follow-up: 39.1% in MT, 31.8% in FWT. Adherence: 91.1% in FWT, 83.9% in MT
Kemmler, 2020 [18]	To assess the effects of dynamic resistance training (DRT) on osteopenia and sarcopenia parameters in community-dwelling men aged 72 years and older with osteosarcopenia.	Body Composition: Assessed using Dual-Energy X-ray Absorptiometry (DXA) for sarcopenia and bone mineral density (BMD) Sarcopenia Parameters: Muscle mass and strength Osteopenia Parameters: BMD at lumbar spine and total hip. (*)	BMD in Lumbar Spine: CG: Decreased by 2.5%.—EG: Increased by 1.6% (non-significant) (*p* = 0.121). Overall significant effect after 54 weeks. SMI: EG: Increased by 3.6% (significant)—CG: Decreased by 1.2%. Significant difference between groups (*p* = 0.671). BMD in Total Hip: CG: Decreased by 1.2%; EG remained stable. o Borderline. No significant difference between groups (*p* = 0.064) Hip and Leg Extensor Strength: EG: Increased by 27%.; CG: Slight decrease. Significant difference between groups (*p* = 0.368). Compliance and Adherence: The exercise group (EG) had 95% compliance with high adherence to protein, calcium, and vitamin D, but 25OHD levels remained below recommended levels despite increases in both groups.
LaStayo, 2017 [19]	To investigate the effects of a multi-component exercise, fall reduction program (MCEFRP) on reducing fall risk in older adults.	**Physical Performance**: Measured through gait speed and other assessments Mini-Cog™ instrument for dementia screening, CSA. **Follow-Up Assessments:** Conducted at pre-MCEFRP (0 months), post-MCEFRP (3 months), and follow-up at 9 and 12 months. (*)	**Mobility**: 3-month improvement in walking distance for both groups: +17.23 m. No group differences **(*p* = 0.604).** **Balance confidence**: Increased at 3 months (RENEW and TRAD (*)): +6.39 points. No group differences **(*p* = 0.160)** **Falls**: 239 days without falls (RENEW) vs. 250 days (TRAD). No differences. (***p* = 0.565)** **Adherence**: RENEW 13% dropout, TRAD 6%. (No *p*-value provided for this variable) **Leg strength**: Increased by 12.94% in 3 months. No group differences. **(*p* = 0.139)**. **Thigh lean tissue**: Decreased at 9 and 12 months. No group differences. **(*p* = 0.930)**.
Mieszkowski, 2018 [20]	To assess the effects of Nordic Walking (NW) combined with different doses of Vitamin D supplementation on body composition, postural control, muscle strength, and functional performance in elderly women.	**Blood Collection and Analysis.** **Body Composition:** Bioelectrical impedance analysis. **Muscle Strength:** Measured peak torque for knee and elbow joints using the Biodex System **Postural Control Assessment:** With the AccuGait platform, evaluating static balance while recording velocity and ellipse area metrics.	**HI-NW (High Intensity Nordic Walkin) Group:** CoP velocity reduced by 18% in HD group post-training (***p* = 0.0372**). **MI-NW (Moderate Intensity Nordica Walking) Group:** 11% reduction in CoP velocity after training (***p* = 0.0089**). **Area 95 (SL Trial):** HD group showed a 64% increase (***p* = 0.0229**) **Eyes Open Trial (EO):** Supplementation had a significant effect on CoP* velocity in the HI-NW group. **Eyes closed Trial (EC)** No significant changes in postural control for both groups.
Mile, 2021 [21]	To assess the effect of a 6-month regular functional exercise training program on patients with sarcopenia, focusing on improvements in muscle strength, body composition, and physical performance.	**Body Composition Assessment** (A) **Short Physical Performance Battery** (SPPB) (*) **Handgrip Strength Measurement** (*) **Posture Characterization Delmas Index** (*) Cobra Test (*) Occiput Wall Distance Test (*) Schober Test (*)	**Body Weight & BMI:** Minor reductions in both groups. **SPPB Scores:** Significant improvement in both groups **(*p* < 0.0001)**. **Lumbar Spine Flexion & Extension:** Significant increases in both groups, **(*p* < 0.0005 to *p* < 0.0001)**. **Posture:** Significant improvement in both groups, (***p* < 0.0001)**. **Muscle Mass:** Increased in both groups, **(*p* < 0.0300 to *p* < 0.0001)**. **Fat Mass:** Decreased in both groups **(*p* < 0.0012 to *p* < 0.0001)**. **Handgrip Strength:** Significant gains in both hands across both groups. **Right Hand:** Both groups showed a slight increase in CG: +1.0 kg, ACEi: +2.0 kg. **Left Hand:** Both improved slightly CG: +1.0 kg, ACEi: +1.0 kg.
Minett, 2020 [22]	To assess the impact of different exercise and nutrition interventions on dietary intake, anthropometrics, body composition, muscle density, intramuscular adipose tissue (IMAT), muscle function, and mobility measures	**Dietary Intake:** 24-h recall and TFEQ. (*) **Anthropometry:** Height (cm), weight (kg), waist circumference (cm). **Body Composition:** DXA for total mass and percentage of fat. **Muscle Density:** pQCT for CSA and IMAT. (*) **Muscle Function:** TUG, gait speed, chair-rise time, and mechanography tests.	**Exercise Groups:** Both WALK and WALK + EX groups significantly improved in the TUG test, gait velocity, and chair-rising test, with no significant differences between groups **(*p*< 0.05).** **Force Plate Mechanography:** The WALK + EX group showed a significant increase in force efficiency **(*p* < 0.05)** and a greater improvement in chair-rise power compared to WALK **(*p* = 0.04).** **Per-Protocol Analysis:** The WALK + EX group had a significant improvement in TUG **(*p* = 0.05)** and a borderline significant increase in chair-rise power **(*p* = 0.07).** The improvement in force efficiency was significantly greater in WALK + EX compared to WALK **(*p* = 0.02).** **Nutrition and Anthropometrics:** The WALK group showed an increase in lean mass, a decrease in carbohydrate intake and fat mass, and a reduction in waist circumference.
Nayasista, 2022 [23]	To analyze the effect of combined locomotor training and aerobic exercise on muscle strength in elderly individuals with locomotive syndrome stage 1.	**Handgrip Strength:** Measurement on the non-dominant hand. Elbow flexed at 90°. Participants instructed to grip the handlebar as hard as possible for 3 s. Three trials performed with a 1-min rest between each. The best value from the three trials was recorded for analysis.	**Handgrip Strength (HGS):** **Treatment Group (TG):** Significant increase in handgrip strength from 13.89 ± 5.27 to 19.06 ± 4.54 (*p* < 0.001). **Control Group (CG):** Non-significant increase from 11.27 ± 2.17 to 13.03 ± 2.54 (*p* = 0.070). o **ΔHGS:** TG exhibited a change of 5.17 ± 1.39, significantly higher than **CG’s** change of 1.76 ± 2.07 **(*p* < 0.001**). o **Geriatric Locomotive Function Scale (GLFS)**. **TG:** Significant improvement from 8.20 ± 1.93 to 6.30 ± 1.64 **(*p* = 0.030)**. **CG:** Significant deterioration from 8.50 ± 1.58 to 10.80 ± 3.19 **(*p* = 0.011)**. o **ΔGLFS:** TG showed −1.90 ± 2.33, while CG presented +2.30 ± 2.26 **(*p* = 0.001)**
Rodrigues, 2022 [24]	To assess the impact of a Muscular Strength Exercise (MSE) program on postural stability and fall risk in octogenarian residents of nursing homes, and to determine the feasibility and effectiveness of the exercise intervention compared to a control group.	**Postural Stability and Fall Risk Assessment:** **Instrument:** Physiosensing^®^ Fall Risk test (Force platform). **Conditions Tested:** o Comfortable stance with eyes open (CSEO)/with eyes closed (CSEC) o Narrow stance with eyes open (NSEO)/with eyes closed (NSEC) **Outcome Measures:** o Speed index (displacement velocity of the center of pressure normalized by height) o Composite speed index score. **Intensity Measurement:** Rate of Perceived Exertion (RPE) scale and heart rate monitoring. **Anthropometric Assessment:** Portable scale (Seca 770), Stadiometer. **Measures:** Body mass, height, and Body Mass Index (BMI)	**BMI:** No significant changes observed at POST16, POST24, or POST40 ***p* = 0.477**. **Composite Index:** **Group Effect:** MSEG had a significantly lower composite index compared to CG (F1,25 = 7.80, ***p* = 0.010**). **Moment Effect:** Significant changes over time **(F3,399 = 15.15, *p* < 0.001)**. **Interaction Effect:** Significant interaction between group and moment (F3,399 = 31.75, *p* < 0.001), with MSEG showing a −2.35 a.u. difference from CG (95%CI [−4.0 to −0.7], ***p* = 0.010**). **Delta Changes:** o POST16 vs. PRE: Δ = −2.6, 95%CI [−3.3 to −1.9], ***p* < 0.001**. o POST24 vs. PRE: Δ = −2.4, 95%CI [−3.0 to −1.7], ***p* < 0.001**. o POST40 vs. PRE: Δ = −2.9, 95%CI [−3.6 to −2.3], ***p* < 0.001**. **Trend:** MSEG consistently had a lower composite index, while CG progressively increased over time. **Postural Control:** Significant differences between groups (F1,25 = 6.44, ***p* = 0.018**) and across moments and conditions (F3,375 = 9.16, ***p* < 0.001),** especially in eyes closed conditions (CSEC, NSEC).
Timon, 2021 [25]	To analyze if strength training under moderate normobaric hypoxia improves functional fitness and reduces fear of falling in healthy older adults, without negatively affecting balance.	**Postural Stability and Fall Risk Assessment** Fall Risk test with Specialized force platform (Physio-sensing^®^ v.19002) **Body Composition:** Dual-energy X-ray absorptiometry (DXA) **Functional Fitness:** Senior Fitness Test (SFT) **Static Postural Control:** Single Leg Stance (SLS) Test o **Conditions:** Eyes open, hands-on hips **Fear of Falling:** Short Falls Efficacy Scale-International (FES-I) questionnaire **Control of Effort During Training:** SpO2%, Heart Rate (HR), Rating of Perceived Exertion (RPE)	**Functional Fitness** **Chair Stand Test**: NT: 12.3 ± 2.2, HT: 12.1 ± 1.5, CON: Not significant. **Significant Difference**: HT vs. CON (*p* = 0.016 *). **Arm Curl Test**: NT: 15.1 ± 4.2, HT: 15.5 ± 5.0, CON: Not significant. **Significant Difference**: NT vs. HT (*p* = 0.004 *). **6-Minute Walk Test**: NT: 554.0 ± 72, HT: 525.9 ± 49, CON: −5.0%. **Significant Difference**: HT vs. CON **(*p* = 0.048)**. **Fear of Falling (FES-I)**: NT: 8.3 ± 1.7, HT: 8.4 ± 1.5, CON: Not significant **(*p* = 0.894). Significant Difference**: NT vs. CON (*p* = 0.037 *). **Body Composition**: NT and HT: No significant changes. CON: Lean body mass: 43.3 ± 10.0 → 42.0 ± 10.2 (*p* = 0.001 *), fat mass: 23.8 ± 5.6 → 25.3 ± 6.0 **(*p* = 0.083)**.
Turunen, 2022 [26]	To investigate whether combined cognitive and physical training offers additional benefits for fall prevention compared to physical training alone in older adults.	**Falls Incidence Rate (IR):** Monitored monthly, recording falls per person-year. **Falls Efficacy Scale International (FES-I):** Questionnaire measuring fear of falling, with scores from 16 to 64. **Injurious Falls:** Falls requiring medical attention, tracked monthly. **Recurrent Fallers:** Participants with 2 or more falls during the study, noted monthly. **Fall-Related Fractures:** Fractures from falls, recorded monthly.	**12-Month Intervention:** o **PTCT:** 132 falls (0.8 falls/person-year) o **PT:** 172 falls (1.1 falls/person-year) o **Difference:** Not significant (IRR = 0.78, ***p* = 0.152)** **12-Month Postintervention Follow-Up:** o **PTCT:** 117 falls (0.8 falls/person-year) o **PT:** 148 falls (0.97 falls/person-year) o **Difference:** Not significant (IRR = 0.83, ***p* = 0.263)** **Falls Efficacy Scale:** o **PTCT:** 3% reduction in concern about falling o **PT:** 4% reduction in concern about falling o **Difference:** Not significant **(*p* = 0.688)**

(*) Gait Velocity Test measures how fast a person walks over a set distance, indicating mobility and balance; Slower speeds can signal mobility issues or fall risk. **Timed Up and Go (TUG) test** measures mobility by timing how quickly a person stands, walks 3 m, and returns to sitting; Slower times indicate potential mobility issues. **Chair Rise Test:** measures lower body strength by timing how quickly a person can rise from a chair multiple times; Slower performance indicates reduced strength or mobility. **One-Repetition Maximum (1RM) test** measures the maximum weight a person can lift on a leg press machine, assessing lower body strength. **30-Second Chair Rise Test (CRT)** counts how many times a person can rise from a chair in 30 s, assessing lower body strength. **10-Meter Walk Test**: Measures walking speed over 10 m to assess gait and mobility. **11-Step Stair-Climbing Test**: Evaluates stair-climbing ability and lower body strength by timing how quickly a person ascends 11 steps. **MCEFRP** multi-component exercise fall reduction program. assesses functional abilities, and rehabilitation needs in clinical settings, focusing on mobility, strength, and overall physical function. **BMD (Bone Mineral Density)** measures the amount of mineral content in bones, indicating bone strength and health. **TFEQ (Three-Factor Eating Questionnaire)** assesses eating behaviors through three factors: cognitive restraint, uncontrolled eating, and emotional eating. **pQCT (Peripheral Quantitative Computed Tomography)** measures bone mineral density and geometry. **CSA (Cross-Sectional Area)** evaluates bone strength. **IMAT (Intramuscular Adipose Tissue)** assesses fat within muscles for metabolic health. **Occiput Wall Distance Test:** Assesses thoracic and cervical spinal deformity (kyphosis) by measuring the ability to touch the wall with the occiput while standing with heels, buttocks, and scapulae in contact with the wall. **Schober Test:** Determines lumbar spine range of motion (flexion) by measuring the distance between two markers on the back before and after forward bending, **Cobra Test:** Evaluates active mobility of the lumbar and dorsal spine by measuring the distance from the incisura jugularis to the support in a maximal push-up position. **Posture Characterization Delmas Index:** Ratio of actual spinal cord length to extended length, categorized into normal, dynamic, and static types. **HGS (Handgrip Strength Measurement):** Utilizes a digital hand dynamometer to measure grip strength in kilograms. **Short Physical Performance Battery (SPPB):** Assesses mobility, balance, and strength through tests like walking speed, static balance, and chair rise. **Test:** Physiosensing^®^. **Fall Risk test** assesses an individual’s likelihood of falling through balance assessments, gait analysis, and strength measurements; helping identify those at higher risk and guiding safety interventions. **Overall Stability Index (OSI)** measures a person’s balance by assessing their postural sway in multiple directions on an unstable surface. Lower scores indicate better stability, while higher scores suggest reduced balance control. **Senior Fitness Test (SFT)** evaluates physical fitness in older adults through assessments like the Chair Stand Test, Arm Curl Test, 6-Minute Walk Test, and Sit-and-Reach Test; helps to tailor exercise programs. TRAD (Traditional resistance exercise. **RENEW** (resistance exercise via negative, eccentrically- induced, work). **CoP** Center of Power. * Significant differences within-group (Pre-Post).

## Data Availability

Data are contained within the article.
